# Supplementary material for: H3K4 methylation regulates development, DNA repair, and virulence in Mucorales
Source: IMA Fungus. 2024 Mar 14;15:6. doi: 10.1186/s43008-023-00136-3 (PMC10938801; doi:10.1186/s43008-023-00136-3)
Supplement: Supplementary file 1 — Additional file 1. Table S1. M. lusitanicus strains used in this study. Table S2. Primers used in this study. Table S3. Set1 proteins used for phylogenetic analysis. Fig. S1. set1 knockout strains of M. lusitanicus are sensitive to SDS, EMS, and UV. Fig. S2. Phenotypic analysis of set1 knockout strains during the interaction with mouse macrophages (J774A.1) [file 43008_2023_136_MOESM1_ESM.pdf]

Table S1. ***M. lusitanicus*** **strains used in this study.**

**Name**	**Genotype**	**Origin**
MU402	*pyrG*$^-$, *leuA*$^-$	(1)
MU636	*leuA*$^-$	(2)
*set1-3*Δ (MU1350)	*set1*Δ*::pyrG*$^+$, *leuA*$^-$	This work
*set1-4*Δ (MU1350)	*set1*Δ*::pyrG*$^+$, *leuA*$^-$	This work

References

1. Nicolás FE, de Haro JP, Torres-Martínez S, Ruiz-Vázquez RM. 2007. Mutants defective in a *Mucor circinelloides* dicer-like gene are not compromised in siRNA silencing but display developmental defects. Fungal Genet Biol 44:504–516.

2. Navarro-Mendoza MI, Pérez-Arques C, Panchal S, Nicolás FE, Mondo SJ, Ganguly P, Pangilinan J, Grigoriev I V., Heitman J, Sanyal K, Garre V. 2019. Early diverging fungus *Mucor circinelloides* lacks centromeric histone CENP-A and displays a mosaic of point and regional centromeres. Curr Biol 29:3791-3802.e6.

Table S2. Primers used in this study.

**Primer name**	**Sequence**	**Experiment**
Set1-UF	CGACTGACACCGTTTCGAAAC	PCR validation
Set1-DR	GAACGTGCTGTATTGTCGCA	PCR validation
set1Rv3	GGCGTCAAGGTGGTTGTTAC	*set1* deletion construct
set1Fw3-pyrG	**CGATAGCATGGCCAGTGTAC**GGTCGCTTTTGTGTGGGG	*set1* deletion construct
set1Rv5-pyrG	**CAAGTACCAATGCTGAGGCA**TCCATGCCCACCACCAAAC	*set1* deletion construct
set1Fw5	GCAGAAGCCACTACCTAAGC	*set1* deletion construct
pyrGFow	TGCCTCAGCATTGGTACTTG	selective marker amplification
pyrGRev	GTACACTGGCCATGCTATCG	selective marker amplification

Table S3. Set1 proteins used for phylogenetic analysis.

Name	Specie	ID FungiDB	ID JGI	ID NCBI
Nc	*Neurospora crassa* OR74A	NCU01206		
Fg	*Fusarium graminearum* PH-1	FGRAMPH1_01G24837		
An	*Aspergillus nidulans* FGSC A4	AN5795		
Ca	*Candida albicans* SC5314	C1_00960C_A		
Sc	*Saccharomyces cerevisiae* S288C	YHR119W		
Ml	*Mucor lusitanicus* CBS 277.49		137855	
Rm	*Rhizopus microsporus* ATCC11559		286187	
Ao	*Apophysomyces ossiformis*			KAF7725527
Ar	*Amylomyces rouxii* NRRL 5866		625830	
At	*Arabidopsis thaliana*			OAP08766

Figure S1

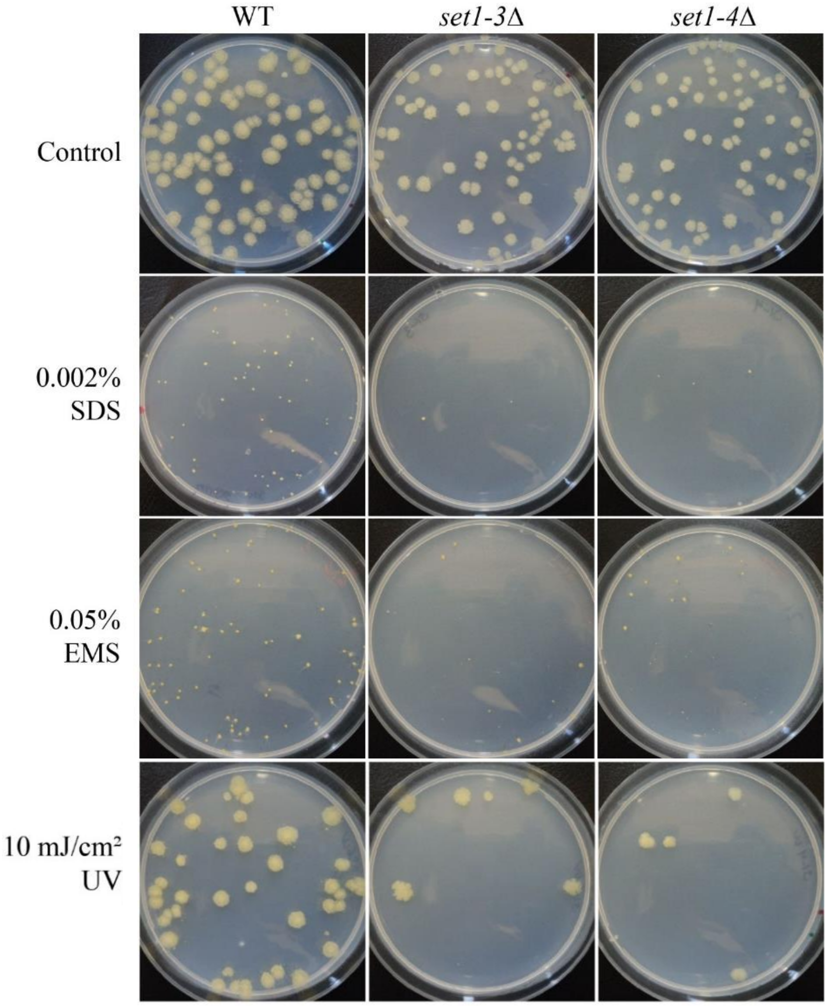

Fig. S1 *set1* knockout strains of *M. lusitanicus* are sensitive to SDS, EMS, and UV. Colonies of MU636 (WT), *set1-3*Δ and *set1-4*Δ after 48 hours of growth at 26ºC on YNB plates (Control) and YNB medium supplemented with SDS (0.002%), EMS (0.05%) or after applying a UV pulse (10 mJ/cm^2).

Figure S2

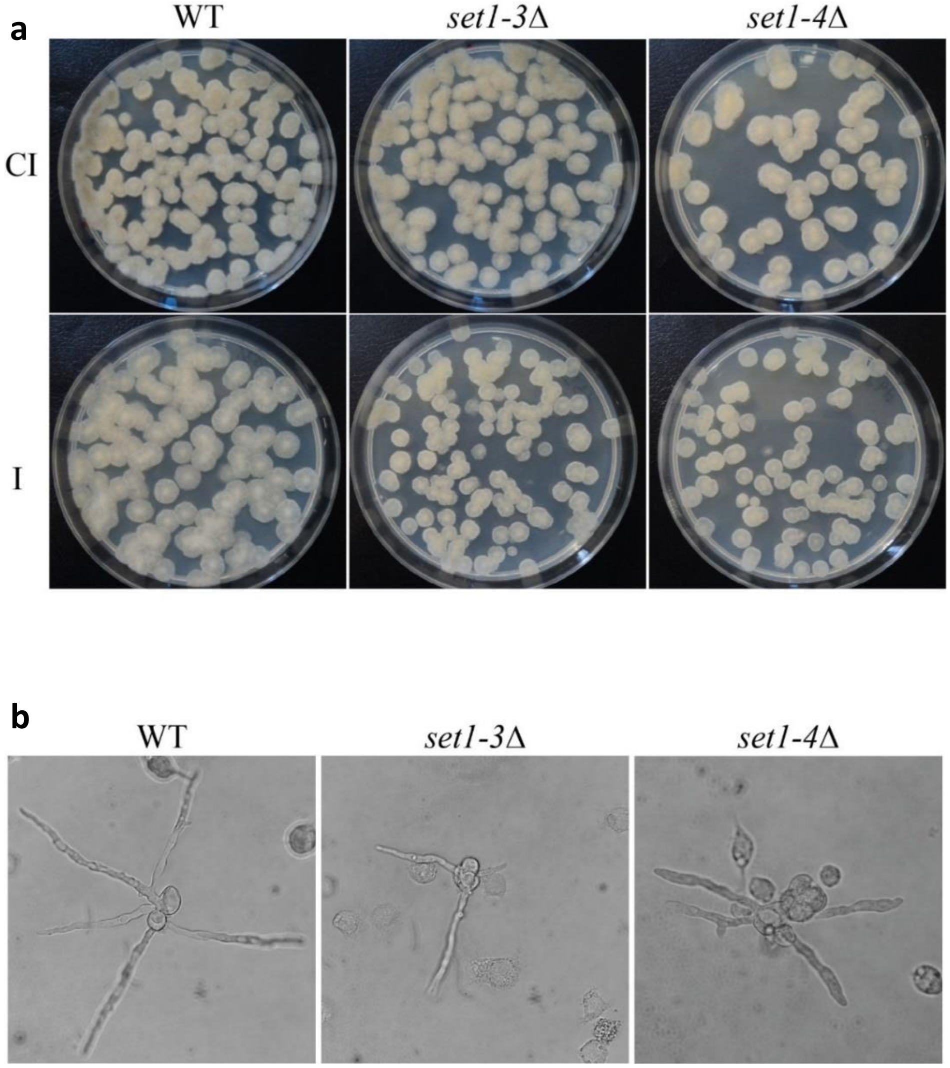

Fig. S2 Phenotypic analysis of *set1* knockout strains during the interaction with mouse macrophages (J774A.1). a Colonies of wild-type strain MU636 (WT) and *set1* deletion mutants developed from spores undergo phagocytosis by mouse macrophages during 5.5 hours of co-culture at 37°C (**I**). The released spores from macrophages were plated on MMC plates to check the growth and colony morphology at 48 hours of culture at 26º C. Spores cultivated under the same conditions but without macrophages served as controls (**CI**). **b** Images show the emergence of the germ tube from spores of WT and *set1*Δ strains phagocytosed by mouse macrophages after 5.5 h of co-cultivation.
